# Influenza Vaccines: A Moving Interdisciplinary Field

**DOI:** 10.3390/v6103809

**Published:** 2014-10-09

**Authors:** Michael Schotsaert, Adolfo García-Sastre

**Affiliations:** 1 Department of Microbiology, Icahn School of Medicine at Mount Sinai, 1468 Madison Avenue, New York, NY 10029, USA; E-Mail: Michael.Schotsaert@mssm.edu; 2 Global Health and Emerging Pathogens Institute, Icahn School of Medicine at Mount Sinai, 1468 Madison Avenue, New York, NY 10029, USA; 3 Division of Infectious Diseases, Department of Medicine, Icahn School of Medicine at Mount Sinai, 1468 Madison Avenue, New York, NY 10029, USA

**Keywords:** influenza, vaccines, live-attenuated virus, adjuvants, correlates of protection

## Abstract

Vaccination is by far the most effective way of preventing morbidity and mortality due to infection of the upper respiratory tract by influenza virus. Current vaccines require yearly vaccine updates as the influenza virus can escape vaccine-induced humoral immunity due to the antigenic variability of its surface antigens. In case of a pandemic, new vaccines become available too late with current vaccine practices. New technologies that allow faster production of vaccine seed strains in combination with alternative production platforms and vaccine formulations may shorten the time gap between emergence of a new influenza virus and a vaccine becoming available. Adjuvants may allow antigen-sparing, allowing more people to be vaccinated with current vaccine production capacity. Adjuvants and universal vaccines can target immune responses to more conserved influenza epitopes, which eventually will result in broader protection for a longer time. In addition, further immunological studies are needed to gain insights in the immune features that contribute to protection from influenza-related disease and mortality, allowing redefinition of correlates of protection beyond virus neutralization *in vitro*.

## 1. Introduction

Influenza viruses cause infections of the human respiratory tract and are responsible for many hospitalizations, deaths and economical losses every year [1], and this despite the fact that influenza is a vaccine-preventable disease. Influenza vaccination is especially recommended for the most vulnerable part of society, *i.e.*, young children, the elderly, pregnant women and immunocompromised individuals. Neutralizing antibodies targeting hemagglutinin (HA), the main surface antigen of influenza virus, correlate with protection [2]. However, the antigenic dress of influenza viruses changes continuously [3] due to the selection of variants generated by the virus’ low fidelity replication machinery. This drift in surface epitopes contained in HA allows the virus to escape antibodies induced by previous infection or vaccination and appears to be driven not only by loss of binding to pre-existing antibodies, but also by changes in receptor binding avidity of the HA [4]. Antigenic drift necessitates updates of currently licensed vaccines for every new influenza season so that the vaccine antigenically matches circulating strains according to predictions sent out by the World Health Organization (WHO). Moreover, completely new influenza viruses can emerge when RNA segments of different influenza strains are re-shuffled in a shared host. These unpredictable events can result in a dramatic antigenic shift leading to a pandemic virus that spreads fast in the human population, as the majority of people lack pre-existing immunity to the new virus. 

In addition, avian influenza viruses like H5N1, H7N9, and H10N8 sporadically cross the species-barrier and infect humans. Such rare zoonotic events often come with high case fatality rates in humans. Although these zoonotic viruses have poor human to human transmission capacity [5], the main part of the population is immunologically naïve and, therefore, at risk in case these viruses acquire the ability to spread. Retrospective analysis after the 2009 influenza pandemic showed that with current technologies an influenza vaccine against a pandemic influenza virus becomes available with a time-delay of about six months, *i.e.*, after the peak of the outbreak [6,7,8]. Analysis of the current vaccine production practices and regulatory measures allows to delineate the constraints in providing efficient influenza vaccines in sufficient amount in a timely manner. In this review we will discuss how improvements in vaccine production and vaccine breadth, as well as in vaccine potency testing and definition of correlates of protection to comply with regulatory issues can significantly contribute to timely influenza vaccine availability in the future.

## 2. Current Influenza Vaccines 

Current licensed vaccines are normalized for the HA antigen of two influenza A strains (H1N1 and H3N2) and the HA of one or two B virus strains (Victoria and Yamagata lineage). The World Health Organization predicts what viruses will circulate in the coming season and provides seed strains for vaccine production each year. Inaccurate predictions may result in reduced vaccine efficacy and effectiveness due to a mismatch between vaccine and circulating strains [9,10,11]. As an example, we cite Belongia and colleagues who compared antigenic match between vaccine strains and influenza virus isolated from patients upon clinical encounter with acute respiratory illness during three subsequent seasons [12]. All patients were considered vaccine targets based on age or high-risk medical condition. Only 5% of patient-derived viruses antigenically matched vaccine strains during the influenza seasons of 2004–2005 and 2005–2006, whereas 91% of viruses recovered from patients matched vaccine strains in 2006–2007. Using data from study participants that tested negative for influenza in the same study, this resulted in a calculated vaccine effectiveness of 10%, 21%, and 52% for the three seasons, respectively.

For influenza A viruses, seed strains typically consist of the HA and NA of seasonal circulating influenza viruses and the internal proteins of a strain that grows to high titers on eggs. These reassortant viruses are in most cases still made by classical reassortment, which is time consuming. More advanced techniques like reverse genetics and synthetic viruses might shorten the generation of 6:2 reassortant seed vaccine viruses drastically [13,14].

Nevertheless, production of influenza vaccines on embryonated eggs requires egg-adaptation of the seed strains containing new HA and NA genes from circulating strains. This might result in mutations that also affect the antigenic properties of HA [15]. Growing virus in cell culture [16,17] may also affect the HA molecule [18], but egg-free production methods will avoid adverse reactions in individuals with allergy to eggs. Moreover, cell culture-based production techniques are easier to scale up in times of shortage of embryonated eggs, e.g., during a pandemic. 

Before seed strains are sent to vaccine manufacturers, potency of vaccine strains is estimated by the single radiation immune diffusion assay (SRID) [19]. The SRID assay is considered one of the most time consuming steps before seed strains are released and shows high interlaboratory variability [20]. More up to date alternatives for the SRID assay have been proposed and need to be evaluated [7,20]. In Europe, additional clinical studies with the candidate seed strains are required, which delays bringing vaccines to the market even further [21].

Once seed strains have been sent out, vaccine manufacturers have the starting material and information for vaccine production. Conventional influenza vaccines consist of inactivated or live attenuated influenza viruses (LAIV). Inactivation is done by treatment with formalin or β-propiolactone (whole inactivated virus, WIV). Dissociation with a nonionic detergent will result in split virus that can be enriched for HA. Whole inactivated virus is more immunogenic compared to split vaccine [22], probably because the remaining viral RNA adjuvants the immune response to the vaccine. Whereas vaccination with egg-derived WIV used to be associated with more adverse reactions, like fever, cell culture-grown WIV comes with similar safety records as split and subvirion vaccines [17]. Live attenuated influenza viruses currently used are so-called cold-adapted influenza viruses that replicate in the upper respiratory tract at temperatures below 37 °C [23]. LAIV are reported to induce systemic humoral antiviral responses less efficiently compared to inactivated virus, but the advantage of replicating virus is their superior induction of mucosal and cellular immune responses against the virus [24,25]. Antiviral T cells may contribute to protection against new influenza viruses, e.g., by providing bystander help to B-cells or by eliminating infected cells [26,27]. The induction of local immune responses by LAIV is associated with virus-specific IgA production in the nasal mucosa, the viral entry gate to the human respiratory tract [28]. Therefore, other immune parameters than hemagglutination inhibition (HAI) by serum may be better correlates of protection for LAIV. 

Other paths than adaptation to growth at lower temperatures have been followed in order to find a strategy to control virus replication of live vaccine strains in the host. Good candidate LAIV should have low chance to revert to a virulent form, *i.e.*, be genetically stable, should grow to high titers in cell culture but have restricted or deficient replication in the host and still induce protective antiviral immunity. Truncation of the viral non-structural protein (NS) 1 protein cripples the virus capacity in countering host interferon responses, which results in reduced replication in the respiratory tract but does not impair the induction of antiviral immunity [29]. Cell culture-derived influenza viruses lacking the NS2 protein are attenuated as well and still induce protective immune responses when used as vaccination tool [30]. Mutations in the ion channel or cytoplasmic tail of the influenza matrix 2 (M2) protein also results in a virus that grows well in cell culture but shows reduced replication *in vivo* and still is immunogenic [31,32].

## 3. Recombinant Vaccines, Virus-Like Particles, Viral Vectors and Genetic Vaccines

The need for a seed strain can be bypassed by making use of recombinant production platforms. Both main influenza surface antigens, HA and NA, have been expressed in bacterial, yeast, insect, plant and mammalian cells as soluble recombinant proteins and were able to induce protective immunity in animal models [33,34,35,36,37,38]. Recombinant soluble influenza proteins have already been tested in clinical studies for different age groups [39,40,41]. Although production of plant-based vaccines may not be as fast as cell culture based production methods, it opens perspectives for immunization via the peroral and gastrointestinal tract, e.g., through the forage of livestock and poultry. 

An alternative for single soluble influenza protein vaccines are virus-like particles (VLPs). VLPs consist of structural virus proteins like the influenza matrix protein which mimic virion configuration. Therefore, they can provide a scaffold for presentation of immunogens like HA and NA but cannot replicate as they lack the viral genome. They are safe and efficient inducers of, potentially even broader [42], protective immune responses. Influenza VLP vaccines for *i.e.*, H5N1, H1N1, and H7N9 produced in different platforms have entered clinical trials [43,44,45]. Similar to VLPs, viral vectors carrying influenza antigens are of interest. Especially the recombinant modified vaccinia virus Ankara (MVA) vector has a very good safety profile in humans and preclinical studies with MVA-based pandemic influenza vaccines are promising [46,47]. An adenovirus vector-based pandemic H5N1 vaccine tested in a clinical study in humans showed that this type of vaccine may especially be of interest for use with poorly immunogenic vaccines in a prime boost setting in which the adenovirus-based vaccination is followed by a parenteral booster injection with inactivated vaccine [48]. 

Because of their fast production capability, genetic vaccines would be good candidate influenza vaccines. However, until today no DNA or RNA-based influenza vaccines have made it to the market. Recently, promising results were communicated for an alphavirus-based H7N9 vaccine, based on delivery of self-amplifying RNA using lipid nanoparticles [49]. After positive validation in humans, this new technology would allow to produce a synthetic vaccine in eight days after sequences of a new emerging influenza virus are available.

## 4. The Role of Vaccine Adjuvants in Induction of Innate and Adaptive Immune Responses 

Administration of a vaccine antigen as such will not always induce the desired amount or type of immunity to the pathogen. Vaccine adjuvants are typically used to help shape the type and size of immune reaction to a certain amount of antigen. Adjuvants may act as a depot and stabilizer for vaccines. Adjuvants can also render antigens more immunogenic by stimulating innate immune responses, which, in turn, will affect the quality of the subsequent adaptive immune response to the vaccine antigen. This may reflect in enhanced and more protective antibody responses induced by a certain amount of antigen. Therefore, use of adjuvants accepted for human use like aluminum salts (aluminum hydroxide, aluminum phosphate and aluminum sulphate) or the squalene-based AS03 and MF59 adjuvants can result in influenza antigen dose-sparing [50,51,52,53,54] as you need less antigen to induce a certain antibody level. Antigen-sparing is important in times of vaccine scarcity, e.g., during a pandemic. 

The mechanisms by which vaccine adjuvants work form an interesting research field that comprises both innate and adaptive immunity. Aluminum salts can induce release of the innate cytokine IL-1β from macrophages through activation of the NOD-like receptor family, pyrin domain containing 3 (NRLP3) inflammasome [55]. Cytokines of the IL-1 family are important for activation of DCs, antigen presenting cells at the interface of innate and adaptive immunity that are responsible for activation of adaptive T cell responses against alum-adjuvanted antigen [56]. Injection of alum also induces release of uric acid, a known activator of DCs [56] that can activate the NLRP3 inflammasome [57]. Uric acid can also activate DCs by direct membrane binding [58] and aluminum salts may also exert their adjuvant effect independently from NLRP3 inflammasome activation. Just as uric acid, adenosine triphosphate (ATP) is released from dying cells and acts like a danger signal capable of activating DCs [59]. The precise mechanism of action for the oil-in-water adjuvants MF59 and AS03 may become clearer in the near future. Interestingly, release of ATP is detected after intramuscular injection of MF59 [60] and the adjuvant effect of MF59 may rely on ASC (apoptosis-associated speck-like protein containing a caspase recruitment domain), but not on the other NLRP3 inflammasome components NLRP3 or caspase-1 [61]. It is likely that oil-in-water adjuvants may directly act on B cells or rely on immune factors other than DCs like macrophages, NKT cells, eosinophils, mast cells or complement for shaping the immune response against the vaccine antigen, as is suggested for aluminum salts [62,63,64,65,66]. 

Injection of alum, MF59 and AS03 results in mobilization of immune cells [60,66,67,68]. Recruitment of monocytes that mature into DCs [56,69,70] results in immune presentation of antigen to T cells. T cells can provide bystander help for B-cells, which promotes class switching and higher antibody titers [71]. It has been shown that aluminum salts and squalene-based adjuvants also promote induction of T cell responses [60,66,72,73]. In general, for influenza vaccines, the oil-in-water vaccines MF59 and AS03 seem to be more potent adjuvants compared to aluminum salts (reviewed in [74]). Further insights in the innate immune mechanisms activated by adjuvants and how this impacts the adaptive immune response to the vaccine antigen will allow fine-tuning of existing and bring forth new candidate adjuvants.

Current inactivated influenza vaccines are typically given intramuscular. The route of vaccine administration can affect the immune answer to vaccination. Induction of antiviral immunity at mucosal sites is interesting as it may stop the virus early in infection but non-live vaccines may require a good adjuvant to initiate adequate immune responses. Initial clinical safety studies for intranasal administration of influenza subunit vaccines formulated with an *Escherichia coli* heat-labile toxin (LT)-based adjuvant did not reveal significant enhancement of adjuvant-associated problems [75,76]. However, despite absence of adverse effects during preclinical and prelicensure studies, inactivated influenza vaccines formulated with LT-based adjuvant are associated with Bell’s Palsy (facial nerve paralysis) upon intranasal vaccine administration during follow up studies, which resulted in withdrawal of the vaccine that was already released for marketing [75,77,78]. This unfortunate event not only highlights the importance of postlicensure surveillance, but also explains the stigma on (LT-based) adjuvants for intranasal administration route. Probably, detoxified forms of LT may still be considered in the future as human mucosal adjuvants for influenza vaccines when administered through routes other than the intranasal one [79]. The advantages of mucosal vaccine administration justify the quest for a safe adjuvant as immune potentiator for this delivery route. Unraveling of the underlying working mechanism and focus on the benefits of adjuvants may help enhancing the community acceptance to vaccine adjuvants in general and for mucosal administration in particular. 

Other adjuvants under consideration are activators of pathogen sensors, TLRs (Toll-like receptors) and RLRs (retinoic acid-inducible gene I (RIG-I)-like receptors), which then induce potent innate responses mimicking those induced by viral infection. As already indicated, whole inactivated influenza vaccines are supposed to be more immunogenic due to the presence of viral RNA, recognized by TLRs and RIG-I. CpG (TLR9 activator) and polyI:C (TLR3 and mda-5 activator) are among some of the adjuvants tested in different preclinical models [69,80,81,82,83,84]. Other adjuvants to be considered are RIG-I activators like the Sendai virus-derived defective interfering RNA [85] and the recently described cGAMP, a cyclic nucleic acid produced upon activation of the cytoplasmic DNA sensor cGAS [86,87].

## 5. Universal Vaccines, T Cells and Correlates of Protection

An influenza vaccine that provides long-term clinical protection would be considered a major amelioration of the current situation. A so-called universal influenza vaccine that protects against drifted or totally different variants of the circulating influenza strains could not only protect against seasonal strains for multiple years but would also reduce the chance of vaccine failure due to an antigenic mismatch between vaccine strains and circulating influenza viruses. Universal vaccines would provide the population with immunological protection in case of an emerging new pandemic influenza virus. Universal vaccines could be stockpiled as they can be given during multiple influenza seasons. 

Universal vaccine development is driven by the observation that immune recognition of conserved epitopes can provide protection against challenge with different influenza strains in animal models [88,89,90,91,92,93,94] and by the finding and characterization of monoclonal antibodies that target conserved epitopes shared by different influenza strains [95,96,97,98]. Virus neutralization, as measured by inhibition of agglutination of avian red blood cells, is the typical correlate of protection for current influenza vaccines. Antibodies that target HA and can neutralize a broad spectrum of influenza A viruses *in vitro* have been characterized [95,96,98,99,100,101,102,103,104,105]. HA-specific monoclonal antibodies with virus-neutralizing capacity are considered useful in a therapeutic setting when administered passively to influenza-infected patients [106]. However, protection in animal experiments was also seen for vaccines and antibodies that provide non-sterilizing, *i.e.*, infection-permissive, immunity. In fact, the protective mechanism of candidate universal vaccines targeting the conserved ectodomain of the matrix 2 protein does not result in virus neutralization *in vitro* but relies on Fc receptors and innate immune components [107,108,109,110] and antibody-mediated protection from influenza A challenge after vaccination with recombinant NP may require the presence of CD8^+^ T cells [90,91]. Broadly neutralizing antibodies that target the HA stalk also rely on Fc receptors for enhanced protection *in vivo* when given at limiting concentration [111]. Neutralizing monoclonal antibodies that target the head of HA tested in the same study do not rely on Fc receptors. Therefore, it is plausible that the* in vivo* reduction of virus titers by broadly reactive HA stalk-specific antibodies induced by vaccination or given prophylactically in the ferret model [92] may fall back on immune mechanisms like antibody-dependent cell-mediated cytotoxicity (ADCC) for the elimination of infected cells when antibody levels are too low in the respiratory tract to directly neutralize the majority of infecting virus by interfering with virus entry. This means that virus neutralization may not be used solely as a correlate of protection for these types of vaccine. Moreover, dependence on Fc receptors implies that the protective effect of some vaccine-induced antibodies is isotype specific. Isotype class switching of antibodies is mainly T cell dependent. These arguments point to the need to define new correlates of protection besides HAI and these should also consider cross-reactive T cells.

T cells typically recognize antigen presented in the context of molecules of the major histocompatibility complex (MHC) upon endogenous expression in or uptake by an antigen presenting cell. This has as a consequence that T cell responses can be primed against more conserved influenza antigens, which may broaden the protective scope of an influenza vaccine [112]. Pre-existing influenza-specific T cell levels have shown to correlate with disease protection in humans in clinical challenge studies [113,114], as well as during the pandemic of 2009 [115]. Cross-reactive memory T cells may interact with naive B cells when drifted influenza virus is encountered [27], and in this way contribute to faster antibody responses to a new influenza strain. Current inactivated vaccines are poor inducers of cross-reactive T cells, a problem that might be overcome in part by the use of adjuvants (see supra and [73,116,117]). Natural infection as well as administration of LAIV or vaccine vectors like MVA expressing influenza antigens will result in endogenous expression of virus antigen. This in turn can result in induction of cross-reactive cellular responses [118,119].

Worries have been voiced that virus neutralization by vaccine-induced antibodies prevents the induction of cross-reactive cellular responses as they prevent or limit virus replication upon virus exposure. This might be of special concern for young children that have never encountered influenza before and therefore are immunologically naïve [120,121,122,123]. On the other hand, vaccine-induced infection-permissive immunity may still be protective and allow virus-induced cross-reactive cellular immune responses after virus encounter that contribute to heterosubtypic protection against reinfection with an influenza virus of a different subtype [124]. An adjuvanted universal vaccine based on a combination of multiple conserved antigens may eventually be considered to further broaden influenza-specific immunity.

## 6. Conclusions and Future Outlooks

Vaccination is still by far the most effective means for preventing disease, including influenza. However, current vaccine practices for influenza leave room for improvement at different levels. Efforts are undertaken for more accurate prediction of strains that will circulate in the next season [125]. The need for yearly vaccine updates together with outdated production techniques and time-consuming regulatory issues are considered major hurdles for adequate vaccine availability in case of a pandemic, leaving the population unprotected until after the peak of virus outbreak. 

Alternative and eventually faster production methods and vaccine types are available. New vaccines, whether or not in combination with adjuvant, may provide broader and possibly long-term protection against influenza. We did not discuss the role of immunity to influenza NA in this review. Nevertheless, this vaccine antigen is very promising as antibodies that inhibit neuraminidase activity correlate with protection in humans and animal models [2,126,127]. Interestingly, antibodies targeting conserved epitopes within the NA molecule may contribute to heterosubtypic cross-protection [127,128,129,130]. NA-specific antibodies do not inhibit virus entry, and, therefore, do not provide sterilizing immunity [126] as provided by HA-specific virus-neutralizing antibodies. New influenza vaccination approaches that do not rely on virus neutralization like those based on NA, M2e or stem HA may require redefinition of correlates of protection. In this regard, both Fc receptor dependent processes, such as ADCC, as well as cross-reactive T cells should be considered. 

A better understanding of the complex interplay between host and pathogen as well as between host and vaccine will allow more rational vaccine design and optimization of vaccination strategies. As of today, systems approaches allow to monitor a plethora of parameters for each individual and induction of innate and adaptive immune responses following administration of an adjuvant or vaccine can be followed rigorously [131,132,133]. In this regard, vaccines and adjuvants are put in the role of immunological research tool that allow to study innate and adaptive immune responses in a controlled setting. Influenza vaccines are given to individuals of most age groups, including those with underlying diseases. In depth multiparametric study of immunological and genetic changes may help predict vaccine outcome and allow definition of better immune correlates of protection. A good understanding of immune correlates of protection will steer further vaccine development and allow optimization of vaccination strategies tailored to the specific needs of specific vaccine target groups.
